# The impact of distance and a shifting temperature gradient on genetic connectivity across a heterogeneous landscape

**DOI:** 10.1186/1471-2148-11-126

**Published:** 2011-05-18

**Authors:** Maurizio Rossetto, Katie AG Thurlby, Catherine A Offord, Chris B Allen, Peter H Weston

**Affiliations:** 1 National Herbarium of NSW, Mrs Macquaries Road, Sydney, NSW 2000, Australia; 2 Mount Annan Botanic Garden, Mount Annan Drive, Mount Annan, NSW 2567, Australia

## Abstract

**Background:**

Inter-population distance and differences in breeding times are barriers to reproduction that can contribute to genotypic differentiation between populations. Temporal changes in environmental conditions and local selective processes can further contribute to the establishment of reproductive barriers. *Telopea speciosissima *(Proteaceae) is an excellent subject for studying the effect of geographic, edaphic and phenological heterogeneity on genotypic differentiation because previous studies show that these factors are correlated with morphological variation. Molecular, morphological and environmental datasets were combined to characterise the relative influence of these factors on inter-population differentiation, and Bayesian analyses were used to investigate current levels of admixture between differentiated genomes.

**Results:**

A landscape genetic approach involving molecular and morphological analyses identified three endpoints of differentiated population groups: coastal, upland and southern. The southern populations, isolated from the other populations by an edaphic barrier, show low migration and no evidence of admixture with other populations. Amongst the northern populations, coastal and upland populations are connected along a skewed altitudinal gradient by genetically intermediate populations. The strong association between temperature and flowering time in *Telopea speciosissima *was shown to maintain a temporally unstable reproductive barrier between coastal and upland populations.

**Conclusions:**

Substrate-mediated allopatry appears to be responsible for long-term genetic isolation of the southern populations. However, the temperature-dependent reproductive barrier between upland and coastal populations bears the genetic signature of temporal adjustments. The extreme climatic events of the last glacial maximum are likely to have caused more complete allochronic isolation between upland and coastal populations, as well as exerting increased selective pressure upon local genomes. However, at intermediate altitudes, current climatic conditions allow for the incorporation of alleles from previously distinct genomes, generating new, intermediate genomic assemblages and possibly increasing overall adaptive potential.

## Background

The identification of reproductive boundaries between genetically differentiated populations can provide useful cues to the factors influencing population-level connectivity and micro-evolutionary processes. Geographic and/or habitat isolation are among the most important factors responsible for establishing the levels of reproductive segregation that lead to measurable genetic differentiation [1]. As dispersal of individuals and gametes is generally more likely among geographically close demes, landscape discontinuities and distributional gaps result in increased neutral divergence [2]. Allochronic separation (divergence in breeding times) can also result in non-random mating and contribute to increased genetic variance across the landscape [3]. A range of environmental factors (particularly temperature and photoperiod) can affect flowering phenology and consequently cause temporal reproductive isolation [4].

Local selective processes can further contribute to the development of pre- or post-zygotic barriers to reproduction. In such circumstances gene flow is restricted by assortative mating caused by the reduced competitiveness of migrants and/or admixed individuals within differentiated habitats [5,6]. Often, more than one factor affects reproductive isolation between populations and to further complicate matters the relative importance of historical processes needs to be taken into account. As a result, the combination of historical and geographical analyses is increasingly used to differentiate between past and present landscape-level connectivity [7].

Temporal changes in local environmental conditions, such as those experienced during the climatic cycles of the Quaternary, can cause localised bottlenecks and extinctions of populations that were previously part of a continuous distributional range [8]. Such periodical contractions can lead to temporal allopatry, drift and vicariant differentiation. As environmental conditions improve landscape-level connectivity can be re-established. These contraction/expansion cycles are influenced by the species' ecology and the landscape features that characterise their habitat, and the genetic structure measured across current-day distributions often reflects the interactions between historical biology and regional geography [9,10].

Unravelling the sequence and tempo of the events establishing reproductive barriers and affecting genetic structure can provide interesting insights into micro- and macro-evolutionary processes, as well as support the development of improved conservation strategies that are mindful of evolutionary potential. This is particularly relevant in a climate change context where predicted climatic shifts could have a significant impact on inter-population connectivity and micro-evolutionary potential.

*Telopea speciosissima *R.Br. (the Waratah, Proteaceae) is a particularly good model for studying the relative influence of climatic and environmental conditions on between-population connectivity: it is distributed along altitudinal and latitudinal gradients; it has a limited flowering season and marked differences in flowering times between populations [11]; and its distribution is interrupted by edaphic barriers. Furthermore, an analysis of morphometric variation in *T. speciosissima sensu lato *[12] showed that what had been considered to be a single, geographically widespread species actually comprised two allopatric, edaphically differentiated taxa: *T. speciosissima *on sandstones of the Sydney Basin and *T. aspera *on the northern granites of the Gibraltar Range (New South Wales, Australia). The major axis of variation from ordinations separated these two species as distinct clusters but secondary ordination axes revealed considerable residual variation, suggesting that differentiation was also present among southern *T. speciosissima *populations.

In this study we aim to detect the genetic signatures that identify the geographic, edaphic and climatic factors leading to differentiation between Waratah populations. In particular, we ask the following questions: is there congruence between morphological and genetic differentiation; how do environmental variables (geographic, edaphic, climatic) differentially impact on genetic structure; and can we use species-wide analyses to identify directionality of gene flow among differentiated population groups, and investigate the temporal strength of reproductive barriers?

## Methods

### Study species

**Figure 1:**
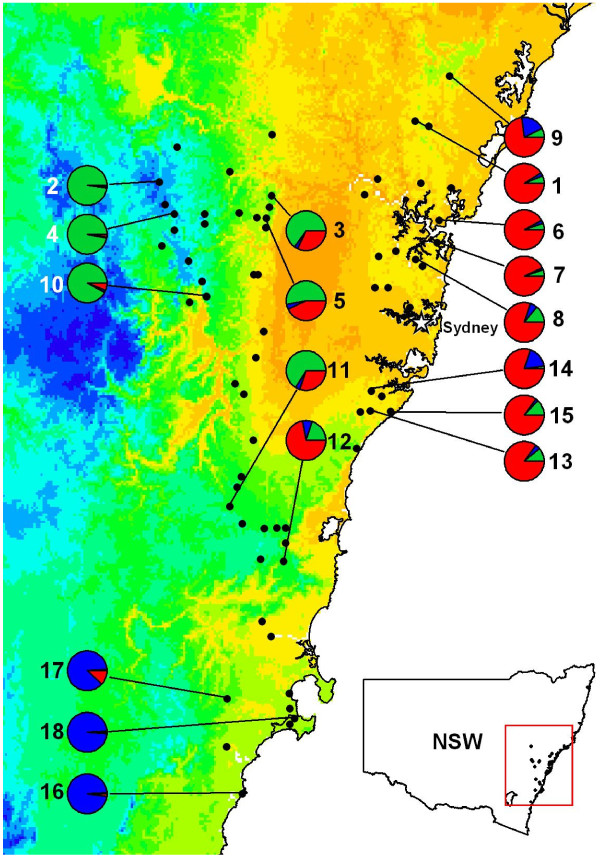
**Distribution of populations and genotypic groups for *T. speciosissima***. Distribution map of *T. speciosissima *(from herbarium records), with sampled population represented by a pie representing Q values for the three main groups identified by STRUCTURE at K = 3 (red: coastal group; green: upland group; blue: southern group). The map includes average maximum temperatures in degree intervals for the month of September (darkest orange: 21-22°C; yellow: 19-20°C; deepest blue: 11-12°C). Population numbers correspond to those listed in Table 1.

**Figure 2:**
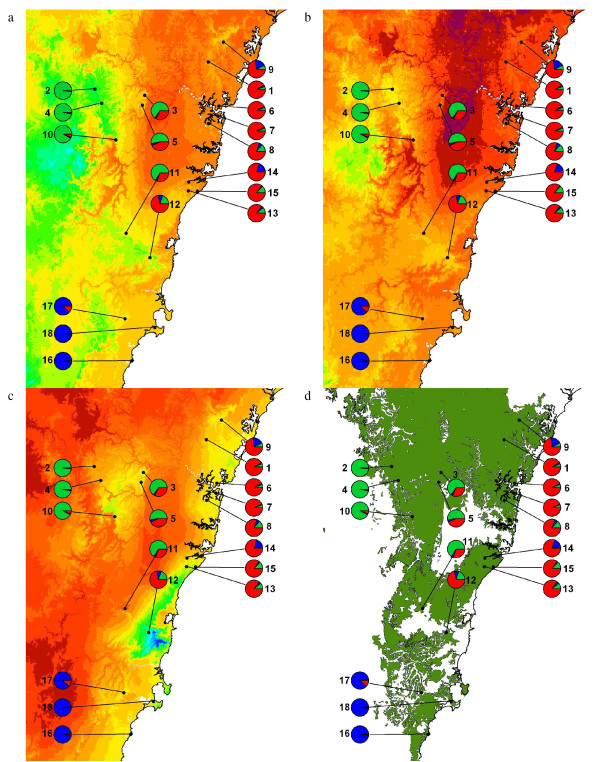
**Environmental maps for *T. speciosissima***. a) Map including average maximum temperatures in degree intervals for the month of October. b) Map including average maximum temperatures in degree intervals for the month of November. c) Map including average annual rainfall in mm (darkest red: 600-700 mm; darkest blue: 1900-2000 mm). d) Map showing the distribution of all sandstone-derived geology types (in green) on which herbarium specimens of *T. speciosissima *are found.

*Telopea speciosissima *occurs sporadically in small populations, of rarely more than a few hundred plants around the Sydney region in south-eastern Australia (Figure 1). Its occurrence coincides with patches of deep, well-drained, acidic, siliceous soils derived from Permian and Triassic Wianamatta sandstones of the Sydney Basin. These sandstones constitute the dominant substrate throughout the Waratah's distribution but in places are overlain by other rocks on which the species does not grow (such as the Triassic shales of the Cumberland Plain; Figure 2d). Agriculture and urbanisation are unlikely to have had a significant impact on current distribution.

Individual plants have long generation times as primary regeneration is through pyrogenesis from large lignotubers. Waratahs flower in a short season, generally lasting six weeks in spring, early in low-lying warmer coastal areas and later in higher cooler areas. The highly conspicuous red inflorescences are primarily bird pollinated [13]. Flowers are protandrous, pollen shedding onto the pollen presenter (modified style tip) several days before receptivity, and have been shown to be exclusively out-crossing [14,15]. Seed production peaks several years after fire regeneration [16]. Seeds have no dormancy mechanisms and germinate readily given adequate moisture conditions [16].

### Sampling, DNA extractions and PCRs

We aimed to obtain a suitable account of the genetic diversity across the entire distribution of the species, as well as obtain a sample that would overlap with that of previous morphological studies [11]. Leaf material was collected and total genomic DNA was extracted using DNeasy^® ^96 plant kits (QIAGEN^®^, Hilden, Germany). Seven simple sequence repeat (SSR) loci specifically developed for *T. speciosissima *[17] were used in this study. Six loci (TS03bgt, TS04bgt, TS12bgt, TS18bgt, TS23bgt, TS27bgt) were dimers (CT repeats) while TS16bgt included a dimer (CT) and a tetramer (CTCA). PCR conditions followed those reported by Porter *et al. *[17]. To test genotyping accuracy PCRs were repeated for over 20% of the reactions. Less than 5% of those repeats identified errors that needed confirmation through replication of PCR and genotyping.

### Genetic diversity

Allelic distribution was measured, and to avoid potential bias caused by uneven sampling [18], a standardized estimate of allelic richness independent of sample size [19] was calculated using the program FSTAT 2.9.3 [20]. Measures of expected (H_e_) and observed (H_o_) heterozygosities, and the inbreeding coefficient (F_IS_; [21]) were also calculated using FSTAT 2.9.3, with the probability of F_IS _being greater than zero determined after 10,000 permutations, and 95% confidence interval determined after 15,000 bootstraps. Hardy-Weinberg and linkage equilibriums were assessed using the exact test with the program GENEPOP 3.2a [22], and significance levels were determined after 1,000 batches of 5,000 iterations each. Sequential Bonferroni corrections were applied to significance tests [23].

Shannon's information index of population subdivision, ^S^*H*_UA_, was used to obtain an estimate of between-population dispersal based on an average effective population size of 100 individuals (*N*_100_m). ^S^*H*_UA _provides a robust estimation of genetic exchange over a wider range of dispersal values than F_ST_, because it is more sensitive to rare alleles, it can incorporate unequal numbers of individuals per population and shows no dependence between measures at different hierarchical levels [24,25].

### Genetic structure

We used the Bayesian clustering method described by Pritchard *et al. *[26] and implemented in STUCTURE 2.3.3 to identify (in the absence of preliminary information on population boundaries) genetically differentiated groups of individuals. After a preliminary test aimed at finding a suitable range for *K *and the optimal burn-in period, we tested *K *from 1 to 18 through five independent runs. All runs were based on 7.5 × 10^5 ^Markov chain Monte Carlo iterations after a burn-in period of 3 × 10^5 ^iterations without prior information on the locality of origin of the individuals sampled. The admixture frequency model was run under the assumption of correlated allele frequencies to improve clustering of closely related populations [27]. The optimal number of clusters was verified using the Δ*K *statistical approach suggested by Evanno *et al. *[28].

Analysis of molecular variance (AMOVA; [29]) was used to quantify variance components and the significance of the genetic subdivisions identified by the Bayesian test, as well as a range of other relevant groupings. Finally, principal coordinate analysis (PCoA) was used to produce a comparative graphical representation of genotypic similarity based on genetic distances among populations. Finally, to reveal the possible influence of isolation by distance (IBD) in explaining between-population genetic differentiation, Mantel tests (999 permutations) were performed between matrices of linearised pairwise F_ST _values (F_ST_/(1- F_ST_) and

ln-adjusted pairwise geographical distances. AMOVA, PCoA, Mantel and ^S^*H*_UA _were run using GenAlEx6.3 [30].

### Between-group admixture

We used the Bayesian model implemented in NewHybrids [31] to detect genetic intermediate individuals resulting from the interbreeding between genetically distinct populations, and distinguish from similarity caused by incomplete lineage sorting. The original tests and simulations by Anderson & Thompson [31] show that admixture can be detected without the need for diagnostic alleles. Although a high number of informative loci produces much better posterior probabilities (PP) for assigning to hybrid categories, when F_ST _>0.2 (as is the case in this study) a smaller number of loci is sufficient for detecting admixture events [32].

Our objective was not to identify and quantify specific admixture categories but rather to ascertain the mixing of differentiated upland and coastal genomes. To do this we conducted a series of preliminary analyses that culminated in a run using seven populations: the three with highest PP of assignment to the upland group, the three with highest PP of assignment to the coastal group, and the four at intermediate altitude. Jeffreys priors were used with a burn-in of 3.5 × 10^5 ^sweeps followed by 7.5 × 10^5 ^sweeps. Posterior probability of assignment as pure, F1s, F2s, and backcrosses were initially measured and than combined to obtain proportions of admixture vs. pure.

### Morphology and phenology

We selected morphometric variables that had high loadings (>|0.95|) on the second axis of the PCoA of Crisp & Weston [11] and scored them for of the available herbarium specimens collected from localities included in our genetic sampling. All variables were measurements or counts of leaf attributes and all were represented by the mean of the measurements or counts from five leaves. We used the same method of ordination used to ordinate our genetic data (PCoA, but using squared Euclidean distances instead of genetic distance). One variable, leaf width, was used to represent size variation. This was also transformed to produce a size-independent shape variable by taking the natural logarithm of the ratio of leaf length to leaf width. Two other variables were similarly transformed.

The date of first flowering of individual plants from six populations across the altitudinal range of *T. speciosissima *(70, 180, 200, 410, 760, 980 m) was recorded during previous site visits (*n*= 256). Flowering dates were converted to day of the year and correlated to altitude of collection site. Data on flowering time of mixed seedlings grown in three plantations at different altitudes in the Sydney region (280, 580, 780 m) were extracted from Dupree & Goodwin [33] and similarly compared (*n *= 731). Finally, flowering date of plants collected from wild sites and grown in a common garden environment (at Mount Annan Botanic Garden, Sydney) was recorded and compared with altitude of provenance (180, 400, 500, 760 m; *n*= 85).

The climatic and environmental data presented in Figures 1 and 2 were created using ArcView V3.3 and ArcGIS V9.2. Temperature and rainfall data are from WorldClim Version 1.4 (release 3) average for 1960-1990. Data are 30 arc-second resolution grid (approximately 0.7 km^2 ^- equivalent to 930 m vertical × 750-775 m horizontal resolution).

## Results

### Genetic diversity in *T. speciosissima*

The seven SSR loci amplified a total of 98 alleles (mean A = 14.0) across the 18 *T. speciosissima *populations. Allelic richness ranged from R_10 _= 3.6 to 6.0 (mean R_10 _= 5.1; Table 1). Overall heterozygosity measures ranged from H_e _= 0.577 to 0.728 (mean H_e _= 0.675), and from H_o _= 0.586 to 0.762 (mean H_o _= 0.659; Table 1), with Kings Tableland being the most and Newnes the least diverse populations (Table 1). Inbreeding coefficient ranged from F_IS _=-0.130 to 0.144, with only three populations being significantly outside HWE before Bonferroni corrections (none after corrections). Turpentine Range and Waterfall Flat showed heterozygotic deficits and West Head heterozygotic excess (Table 1). Species-level inbreeding measures (for 326 individuals across 18 populations) were F_IS _= 0.024 (P < 0.05), F_IT _= 0.148 (P < 0.001) and F_ST _= 0.127 (P < 0.001). Pairwise tests of linkage disequilibrium showed one combination (TS03bgt and TS18bgt) to be out of equilibrium but not after Bonferroni correction.

**Table 1:** Population genetic statistics for *T. speciosissima*

**No**.	Population	Latitude	Longitude	Alt (m)	N	A	**R**_**10**_	**H**_**e**_	**H**_**o**_	**F**_**IS**_	Q Upland	Q South	Q Coast
1	Kulnura	33 13 S	151 12 E	340	21	7.4	5.6	0.670	0.660	0.016	0.06	0.03	**0.91**
2	Newnes Forest	33 24 S	150 13 E	1140	20	4.0	3.6	0.577	0.593	-0.029	**0.98**	0.01	0.01
3	Mountain Lagoon	33 27 S	150 39 E	590	20	6.6	5.5	0.721	0.693	0.040	0.65	0.03	0.32
4	Bells Line of Road	33 30 S	150 16 E	1090	20	5.1	4.4	0.676	0.693	-0.025	**0.97**	0.01	0.02
5	Kurrajong Heights	33 31 S	150 37 E	580	10	4.4	4.4	0.616	0.586	0.051	0.53	0.04	0.43
6	Patonga	33 32 S	151 17 E	180	19	6.1	5.1	0.682	0.669	0.020	0.05	0.03	**0.92**
7	West Head	33 37 S	151 17 E	130	15	5.7	5.1	0.677	0.762	-0.130*	0.05	0.02	**0.93**
8	Duffys Forest	33 39 S	151 11 E	190	22	7.9	6.0	0.701	0.649	0.076	0.14	0.06	0.80
9	Watagan	33 04 S	151 20 E	460	21	6.6	5.7	0.726	0.677	0.070	0.07	0.20	0.73
10	Kings Tableland	33 46 S	150 23 E	810	21	6.9	5.9	0.728	0.748	-0.029	**0.93**	0.02	0.06
11	Mt. Alexandra	34 27 S	150 27 E	750	20	5.6	4.8	0.677	0.686	-0.013	0.66	0.04	0.30
12	Carrington Falls	34 38 S	150 39 E	560	19	6.7	5.7	0.711	0.692	0.028	0.2	0.08	0.72
13	Waterfall Flat (Royal NP)	34 09 S	151 00 E	150	15	6.0	5.4	0.683	0.610	0.111*	0.11	0.04	0.86
14	Bottle Forest (Royal NP)	34 05 S	151 00 E	200	11	6.0	5.8	0.717	0.701	0.024	0.02	0.18	0.80
15	Curra Moors (Royal NP)	34 09 S	151 05 E	80	10	5.0	5.0	0.620	0.586	0.058	0.13	0.02	0.85
16	Ulladulla	35 22 S	150 28 E	40	21	5.3	4.4	0.601	0.612	-0.018	0.01	**0.97**	0.02
17	Turpentine Range	35 04 S	150 25 E	350	21	6.4	5.3	0.697	0.599	0.144**	0.01	**0.87**	0.12
18	Jervis Bay	35 08 S	150 41 E	50	20	5.3	4.6	0.670	0.650	0.030	0.01	**0.98**	0.01

	Mean	-	-	-	18.1	5.9	5.1	0.675	0.659	0.024	-	-	-

*P < 0.05; **P < 0.01 (no F_IS _values were significant after Bonferroni corrections).

Population name, location and population-specific (as well as average) population genetic statistics for *T. speciosissima*. Number of individuals (N), allelic number (A) and richness (R_10_), expected (H_e_) and observed (H_o_) heterozygosities, inbreeding coefficient (F_is_), and average assignment values (Q) to the three groups (upland, southern, coastal) identified by STRUCTURE at K = 3 (in bold the three populations with highest values) are listed.

### Genetic structure in *T. speciosissima*

**Figure 3:**
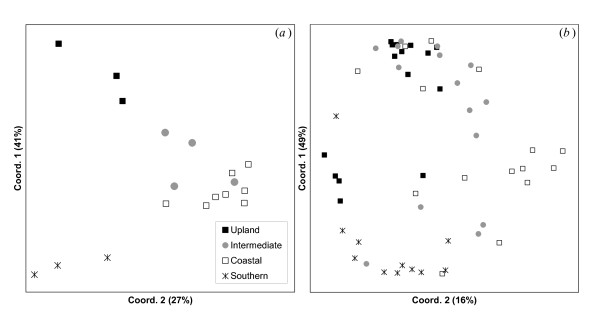
**PCoA of genetic and morphological data**. Plots of the first two principal coordinates obtained for *T. speciosissima *from the principal coordinate analysis of: a) pairwise genetic distances (by population) and b) morphometric distances (by individual).

Bayesian clustering as performed by STRUCTURE produced substantial increases in average *Ln*P(D) values at *K *= 2 and *K *= 3, and the Δ*K *statistic preferentially supported *K *= 3 (Δ*K*_2 _= 127, Δ*K*_3 _= 485). At *K *= 3, populations were grouped into coastal (located within 30 km of the coast), upland (distributed at >800 m altitude and >85 km from the coast) and southern (located south of the Shoalhaven catchment; Table 1 and Figure 1). Three populations from each of these groups registered high coefficients of memberships (Q > 0.85, and two of these in southern and upland groups had Q > 0.95). The four populations located at intermediate altitude (between 400 m and 800 m) had Q < 0.70, except for Carrington Falls (located only 20 km from the coast, Q = 0.72 coastal). The PCoA supported the presence of the three endpoints of genetically differentiated population groups, and placed the populations from intermediate altitude between coastal and upland groups (except for Carrington Falls which was placed with coastal populations; Figure 3a).

AMOVA supported significant segregation between the three population groups. High levels of genetic variance were partitioned to between-group differentiation, although most of the genetic differentiation was partitioned to within-population differentiation (as expected for outcrossing species). Table 2 shows that most of the genetic divergence was caused by the upland and southern groups (average divergence from the other populations: F_ST _= 0.191 and F_ST _= 0.174 respectively). Average genetic divergence between upland and southern population was F_ST _= 0.228, while average F_ST _among all other populations was F_ST _= 0.068. Average pairwise F_ST _values and ^S^*H*_UA_-based estimates of between-population dispersal (based on N_e _= 100) suggest high levels of gene flow within population groups (coastal, upland, southern) and between pairings of upland vs. intermediate, and coastal vs. intermediate populations (Table 2). Low but significant IBD was detected when all 18 populations were included (R^2 ^= 0.236; P < 0.001), but not across the 10 populations used for the NewHybrid analysis (R^2 ^= 0.072; P = 0.07), suggesting that the differentiation measured along the altitudinal gradient was not caused by geographic distance alone.

**Table 2:** Genetic structure and gene flow in *T. speciosissima*

Average pair-wise values	F_ST_	m	AMOVAs	Among Groups	Among Pops	Within Pops
*T. speciosissima *mean	0.127	1.877				
Upland pops	0.079	1.947	Upland vs. rest	16% ^†^	15% ^†^	71% ^†^
Coastal pops	0.056	3.091	Coastal vs. rest	3% ^†^	21% ^†^	76% ^†^
Southern pops	0.068	4.661	Southern vs. rest	15% ^†^	15% ^†^	70% ^†^
Southern vs. rest	0.174	0.812				
Upland and Coastal vs. all others	0.128	0.899	Upland vs. Coastal vs. Southern	25% ^†^	8% ^†^	67% ^†^
Coastal vs. Intermediate	0.079	1.981	Upland vs. Coastal vs. Southern vs. all others	15% ^†^	10% ^†^	75% ^†^
Upland vs. Intermediate	0.112	2.024	Upland vs. Southern vs. all others	18% ^†^	10% ^†^	72% ^†^

^† ^P < 0.001

Measures of genetic structure and gene flow among selected population groupings (as identified by STUCTURE at K = 3). F_ST _and estimated number of migrants (m) derived from N_e _= 100 and derived from ^S^*H*_UA _are presented, as well as relevant percentage of genetic diversity partitioned to within, between and among population groups (AMOVA) and significance values.

### Connectivity and admixture

**Figure 4:**
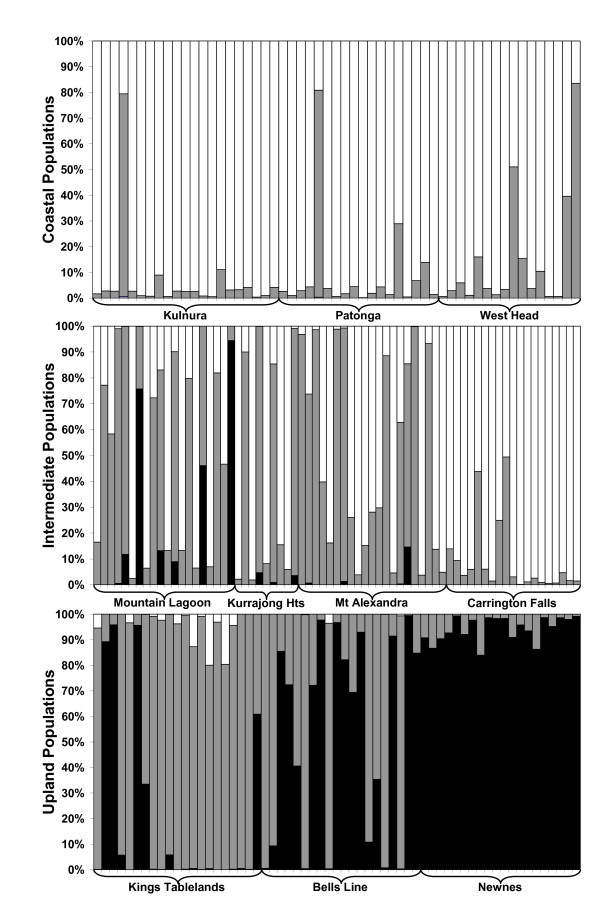
**Proportions of admixture among selected populations**. New Hybrids results showing the relative proportion of admixture among genotypes representative of coastal, intermediate and upland populations. White represents pure coastal source, black pure upland source, and grey admixed origin.

Strong patterns of pure (ie. with PP > 80% of being assigned to one of the three distinct groups) and admixed genotypes were detected across populations (Figure 4). F2 was the most common admixed type with the other types rarely being given cumulative PP of over 1% and never over 5% (results not shown). Previous studies have shown that the presence of a third genome could be a cause of the high frequency of F2s [34] and to test for such possible interference we followed two validation tests. First, we added a third genome to the analysis (by including the three southern populations) to test if that genome could be indirectly biasing our results. However, its inclusion resulted in all individuals from the southern populations being assigned to pure southern and all other individuals being assigned to a second category of pure genotypes, with a complete absence of admixture. Secondly, we sequentially excluded each of the pure genomes (coastal and upland) from the analysis, with this test not resulting in a conversion of F2 individuals into pure. The results of these verification trials provided further support to the admixed origins of individuals scored as F2s. A low frequency of detected F1 has been previously reported in a taxonomically diverse range of taxa and could suggest that hybrid events are relatively rare [35].

A recent study by Roberts *et al. *[36] showed that relaxing assignment threshold to pure from 95% PP to 80% PP made little difference to their findings. As a result, here we define as pure individuals, those with >80% PP of being assigned to a particular population group. In the three selected coastal populations 89% of individuals were assigned to pure coastal. This was in contrast with the three selected upland populations where 51% of individuals were assigned to pure upland. Interestingly, the Newnes population showed no evidence of admixture in contrast to the geographically close Bells Line of Road population in which 35% of individuals had a PP of >80% of being admixed. The situation for the intermediate populations was more complex: a single individual at Mountain Lagoon was identified as pure upland, while 52% of individuals were assigned to pure coastal. At the opposite end of the spectrum all individuals were assigned to pure coastal at Carrington Falls.

### Morphology and phenology

PCoA of the morphometric data produced a similar ordination to the PCoA of genetic data in that specimens from upland, southern and coastal groups of populations formed clusters that occupied the same relative positions to the corresponding clusters in the genetic analysis, albeit with some overlap as would be expected from characters that are both phenotypically labile and ontogenetically variable (Figure 3b; [12]).

First flowering in five wild locations occurred when the 10 day mean maximum was 20°C and the minimum was 9°C (Australian Bureau of Meteorology), regardless of altitude. The timing of the temperature shift necessary for flowering varied across the altitudinal gradient, and corresponded to the genetic differentiation observed between coastal and upland populations (Figures 1 and 2).

The analysis of flowering time from 256 flowering events from seven natural locations distributed along the altitudinal gradient showed a very strong association between altitude and flowering time (*R*^2 ^= 0.929; *P *< 0.0001). For instance, average flowering time at Evans Lookout (980 m) was on Julian day 312, while at the coastal site at Patonga (180 m) it was on day 261. There was a weaker but significant relationship between flowering time and altitude in three cultivated populations sourced from mixed genotypes (*R*^2 ^= 0.471; *P *< 0.0001), and data from the common garden environment suggested that altitude of original collection (sourced from three different altitudes) had no influence on flowering within a controlled environment (*R*^2 ^= 0.011; *P *= 0.174).

## Discussion

### Genetic diversity and structure

Levels of genetic diversity were generally consistent across all sampled Waratah populations, irrespective of their size and location. Heterozygosity is likely to be maintained in small populations through preferential outcrossing (*T. speciosissima *is highly receptive to cross pollen; [37,38]), and measured values were similar to those recorded for other geographically localised, bird pollinated species of Proteaceae (eg. *Banksia hookeriana*; [39]).

Although high gene flow maintains equilibrium at a local scale, between-population connectivity appears to be less consistent in *T. speciosissima*. PCoA and Bayesian assignment identified three major population groups (southern, upland, coastal) supported by AMOVA and between-group F_ST _values. Morphological analysis discovered similar patterns of geographic variation, confirming strong differentiation in the south and identifying a previously unreported upland morphotype.

Understanding the processes leading to genetic and morphological differentiation requires the identification of the barriers that cause partial or complete reproductive isolation [4]. Edaphic conditions and geographic distance are likely to represent important isolating mechanisms for the Waratah. Shale-derived soils of the Cumberland Plain form an island of unsuitable habitat at the heart of the overall distributional range of the species (Figure 2d), and the southern populations are located at the edge of the sandstones to which the species is restricted. Furthermore, the southern group grows on Permian sediments (rather than on Triassic rocks) and is isolated by a barrier of volcanic-derived soils. The combination of geographic distance and marginal habitat could therefore explain the considerable genetic and morphological differentiation detected in the south.

Although the terminally winged seeds of *T. speciosissima *are not easily dispersed and tend to germinate in proximity to the maternal plant [16], it is unlikely that limited dispersal is the only mechanism restricting gene flow. The Waratah is bird and mammal pollinated [13] and our data show that gene flow across large distances is possible (within the coastal group for example), thus suggesting that factors other than dispersal impact on between-population connectivity.

### Altitude, temperature, phenology and local genomes

Our study detected considerable genetic structure along the altitudinal gradient and a close association between altitude, temperature and flowering time in *T. speciosissima*. Flowering in the Waratah is triggered by a shift in average maximum temperature to around 20°C, and this shift occurs gradually along the altitudinal gradient (Figures 1, 2). Coastal populations flower early (20°C average attained in September) and upland populations flower late (20°C average attained in November). The temperature gradient associated with topography impacts on phenology and forms a temporal isolating barrier between coastal and upland populations.

If temporal reproductive isolation is the predominant factor separating coastal and upland genomes we would expect it to be incomplete across transitional zones where flowering overlap is likely. The Waratah populations that are intermediate in altitude and flowering time are also genetically intermediate (Figures 3, 4), suggesting that they operate as a contact zone between the upland and coastal genomes (as also supported by the estimated number of migrants; Table 2).

In contrast, there was very low migration and no evidence of admixture between the southern and any of the other populations, suggesting that the temporal barriers to gene flow along the altitudinal gradient are easier to overcome than those constrained by geographic distance and edaphic factors. While geographic isolation can be difficult to overcome without suitable means of dispersal [40], allochronic isolation can be quickly bypassed following changes in climatic conditions that enable phenological overlap.

### Current vs. historical isolation

Admixture can take place without the need for complete sympatry, particularly when pollen exchange is mediated by highly mobile vectors [1]. As a result, unless the Waratah populations at intermediate altitude have established relatively recently a more even allelic spread should be expected along the East/West distributional axis, especially given the level of migration between the intermediate and the other two population groups. Two main factors could lead to the strong genetic structure observed: long-term fluctuations in climatic conditions that affect the strength of phenological isolation, and adaptation to local conditions that lead to habitat isolation.

If a strong association between flowering time and local conditions exists, a change in these conditions will directly impact on reproductive isolation [41]. Our common-garden phenological data emphasize the predominance of a genetically controlled response to shifting temperature conditions along the altitudinal gradient. As a result, the temperature differential that separates the upland from the coastal genomes represents an adjustable reproductive barrier. In current climatic conditions, geographic proximity and partial phenological overlap allow for genetic exchange to occur and result in the observed admixture front.

However, during glacial maxima temperature gradients were more intense, and in some upland areas temperatures were 10°C lower than present [42]. Hesse *et al. *[43] suggested that during the LGM the Newnes Plateau (where some of the upland Waratah populations are located) experienced extreme climatic conditions. The activity of aeolian sand dunes between 30 and 15Kya suggests that during the LGM (and previous glacial peaks) the area was dominated by grasslands [43]. As a result, surviving *T. speciosissima *populations would have experienced significantly cooler and drier conditions than those experienced by the populations living at lower altitudes, and allochronic isolation would have been stronger or complete. In such climatically distinct areas, assortative mating would have been a powerful factor in developing and maintaining the genomic differences that were measured in this study.

Exposure to differentiated environmental conditions could also have established selective reproductive barriers and reduced the viability of migrant and/or admixed genotypes. The fact that the Newnes population is not admixed (unlike other upland populations) suggests that vertebrate-mediated pollen dispersal is not uniform across what is fairly continuous habitat. Such pattern suggests that habitat isolation might also contribute to the genetic differentiation observed between upland and coastal populations, a hypothesis we are currently testing via cross-pollination, reciprocal transplant and controlled-environment experiments.

## Conclusions

Our study on a long-lived woody shrub distributed along altitudinal and latitudinal gradients, provides evidence for the contrasting temporal stability of different reproductive barriers. While the association between geographic and genetic isolation appears to be stable, the temperature-dependent reproductive barrier between upland and coastal population bears the genetic signature of temporal adjustments. The detection of localised admixture events and the availability of morphological, phenological and climatic (current and historical) information helped us identify a transitional zone established as a result of the post-glacial alleviation of extreme differences in climatic conditions.

These temporal changes resulted in the merger of reproductively isolated and differentiated genomes, a process that can have deleterious (sterility, homogenisation, outbreeding depression) or beneficial (the incorporation of novel diversity favouring adaptation to changing conditions) evolutionary consequences [44]. The long-term consequences of this genomic reshuffling in the Waratah are yet to be clarified but provide a valuable framework for in-depth adaptation studies. Understanding how changes in connectivity driven by climatic fluctuations affect natural populations has important implications for the management of biodiversity in view of predicted environmental change, and will be the focus of long-term in-situ experimentation and genomic studies on the Waratah.

## Authors' contributions

MR and PHW conceived and designed the study; KAGT, CAO, CBA, PHW respectively contributed the genetic, phenological, environmental and morphological data; MR analysed the molecular data and prepared the manuscript with all authors editing and approving the final manuscript.
